# Role of Squalene Epoxidase Gene (*SQE1*) in the Response of the Lichen *Lobaria pulmonaria* to Temperature Stress

**DOI:** 10.3390/jof10100705

**Published:** 2024-10-09

**Authors:** Alfred O. Onele, Moatasem A. Swid, Ilya Y. Leksin, Daniya F. Rakhmatullina, Ekaterina I. Galeeva, Richard P. Beckett, Farida V. Minibayeva, Julia N. Valitova

**Affiliations:** 1 Kazan Institute of Biochemistry and Biophysics, FRC Kazan Scientific Center, P.O. Box 261, 420111 Kazan, Russia; donjay.ao@gmail.com (A.O.O.); moatasem.sowed@gmail.com (M.A.S.); lecsinilya@mail.ru (I.Y.L.); rdf137@mail.ru (D.F.R.); kgu@mail.ru (E.I.G.); rpbeckett@gmail.com (R.P.B.); fminibayeva@gmail.com (F.V.M.); 2 Institute of Fundamental Medicine and Biology, Kazan Federal University, Kremlyovskaya 18, 420008 Kazan, Russia; 3 School of Life Sciences, University of KwaZulu-Natal, Private Bag X01, Scottsville, Pietermaritzburg 3209, South Africa

**Keywords:** ergosterol, heat shock protein, lichen, *Lobaria pulmonaria*, squalene epoxidase, sterols, *Symbiochloris reticulata*

## Abstract

Currently, due to the increasing impact of anthropogenic factors and changes in solar activity, the temperature on Earth is rising, posing a threat to biodiversity. Lichens are among the most sensitive organisms to climate change. Elevated ambient temperatures can have a significant impact on lichens, resulting in more frequent and intense drying events that can impede metabolic activity. It has been suggested that the possession of a diverse sterol composition may contribute to the tolerance of lichens to adverse temperatures and other biotic and abiotic stresses. The major sterol found in lichens is ergosterol (ERG); however, the regulation of the ERG biosynthetic pathway, specifically the step of epoxidation of squalene to 2,3-oxidosqualene catalyzed by squalene epoxidase during stress, has not been extensively studied. In this study, we used lichen *Lobaria pulmonaria* as a model species that is well known to be sensitive to air pollution and habitat loss. Using in silico analysis, we identified cDNAs encoding squalene epoxidase from *L. pulmonaria*, designating them as *LpSQE1* for the mycobiont and *SrSQE1* for the photobiont *Symbiochloris reticulata*. Our results showed that compared with a control kept at room temperature (+20 °C), mild temperatures (+4 °C and +30 °C) did not affect the physiology of *L. pulmonaria*, assessed by changes in membrane integrity, respiration rates, and PSII activity. An extreme negative temperature (−20 °C) noticeably inhibited respiration but did not affect membrane stability. In contrast, treating lichen with a high positive temperature (+40 °C) significantly reduced all physiological parameters. Quantitative PCR analysis revealed that exposing thalli to −20 °C, +4 °C, +30 °C, and +40 °C stimulated the expression levels of *LpSQE1* and *SrSQE1* and led to a significant upregulation of *Hsps*. These data provide new information regarding the roles of sterols and Hsps in the response of lichens to climate change.

## 1. Introduction

There is increasing evidence indicating that climatic conditions are globally changing, posing great threats to biodiversity [1]. As symbiotic organisms, lichens represent an interesting model system for studying how organisms adapt and respond to environmental changes. Despite their ability to survive air-drying, i.e., their poikilohydric nature, lichens are among the most sensitive organisms to climatic factors and often have been used as bio-indicators [2,3].

However, some lichens have a remarkable capacity to survive under extreme stressful conditions, likely due in part to their high stress tolerance when desiccated [4,5]. While the precise mechanisms of heat stress damage on lichens remain unclear [6], it is evident that cell membranes are severely affected. Studies have shown that increasing temperatures leads to a reduction in chlorophyll content [7] and can impact the synthesis of lichen substances, potentially through thermal degradation [8]. Air-dry lichens exhibit significant heat resistance, with air-dried thalli readily tolerating temperatures of 70–101 °C compared with 35–46 °C in hydrated thalli [9]. However, even when air-dried, some species nevertheless can be highly sensitive to moderately high temperatures [10,11,12]. This indicates that heat tolerance varies among lichen species, and it is unclear whether even air-dried forms are universally resilient to heat stress [12,13].

The projected impact of climate change is particularly severe for the old woodland lichen *Lobaria pulmonaria* compared to other species, with forecasts suggesting a reduction in its climatic suitability to only 15% of its current geographical range by 2080 [14]. The mycobiont *L. pulmonaria* is a lichen that exhibits a complex symbiotic relationship with a green algal primary photobiont, *Symbiochloris reticulata* [15], and a minor cyanobacterial partner, *Nostoc* sp. (*Nostocales*) [16]. Moreover, *L. pulmonaria* seems to have evolved powerful molecular pathways to cope with environmental fluctuations and stress, as it can acclimate to new habitats through transcriptomic convergence, meaning that it adjusts its gene expression patterns in response to changing temperature conditions [17]. It was demonstrated that even moderate changes in the temperature can cause significant changes in gene expression. The lichen-forming fungus *L. pulmonaria* upregulated and differentially expressed certain heat shock genes when exposed to sudden temperature increases from 5 to 25 °C [5]. Furthermore, ref. [17] investigated the effects of various factors on gene expression in *L. pulmonaria* and its green algal primary photobiont, *S. reticulata*. The authors found that both symbionts upregulated heat shock genes when faced with unexpected temperature increases. Heat shock proteins (Hsps) and other stress-induced proteins play a crucial role in repairing or removing heat-induced cellular damage by preventing the aggregation of denatured proteins [18,19].

Among multiple mechanisms of tolerance of organisms to adverse environments, sterols play important roles because they are important components of the cell membranes and lipid rafts [20]. Lichens have a unique and diverse sterol composition that differs from that of free-living fungi and algae, and sterols may be at least in part responsible for the high stress tolerance of these symbiotic associations [21,22]. 

**Figure 1:**
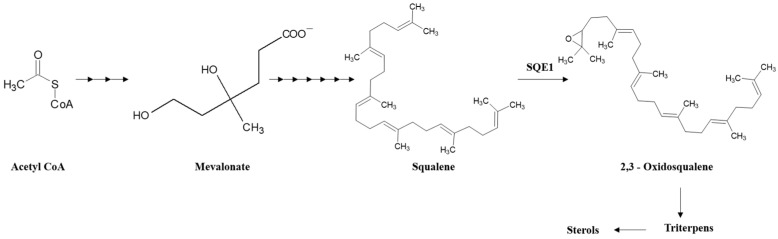
Simplified scheme of sterol biosynthesis including the epoxidation of squalene catalyzed by squalene epoxidase (SQE1).

The genes encoding enzymes involved in the steps of sterol biosynthesis have been intensively studied in various model organisms [23] but not in lichens. An important step is the epoxidation of squalene, which is converted to 2,3-oxidosqualene, catalyzed by the enzyme squalene epoxidase (SQE), also known as squalene monooxygenase (Figure 1). In mammals, SQE is considered as a key enzyme in the cholesterol biosynthetic pathway [24]. In plants, SQE is a rate-limiting enzyme in the synthesis of phytosterols [24,25]. Moreover, plants possess an additional pathway for synthesizing triterpene glycosides (saponins) that branches off from 2,3-oxidosqualene. 

Seedlings of transgenic tobacco containing the squalene gene from *Asparagus racemosus* (*ArSQE*) subjected to abiotic stress display early germination and a generally increased stress tolerance [26]. In yeast, higher levels of egosterol, a molecule synthesized in part by the *SQE1* gene, are associated with increased tolerance to low and freezing temperatures [21,27]. Similarly, ref. [28] reported the upregulation of genes involved in the biosynthesis of ergosterol genes at 25 °C in the Chlorophycean photobiont of *Peltigera britannica*. However, the role of SQE in stress tolerance of lichens has not yet been thoroughly studied. Furthermore, there are currently no data available on SQE in *L. pulmonaria*, and no studies have examined how temperature stress affects the expression of genes encoding the squalene epoxidase protein. 

Therefore, the aim of the present research was to study the changes in the physiology of *L. pulmonaria* in response to low and elevated temperatures and to elucidate the putative role of SQE in the tolerance or sensitivity of the lichen symbionts to temperature stress. In this study, we first measured physiological parameters such as membrane stability, rates of respiration, PSII activity, and the expression of genes encoding heat shock proteins (Hsps) in the thalli of *L. pulmonaria* exposed to freezing, low positive, and elevated temperatures. Secondly, we in silico identified *SQE1* in both the mycobiont *L. pulmonaria* and the green algal photobiont *S. reticulata* and carried out a molecular characterization of their proteins. Thirdly, expression of *SQE1* in both symbionts was analyzed following temperature stress.

This study provides a better understanding of the role of squalene epoxidase and *Hsps* in *L. pulmonaria* under temperature stress conditions. Studying how lichens respond to temperature stress can aid in developing conservation strategies to protect these important organisms and the ecosystems they inhabit. 

## 2. Materials and Methods

### 2.1. Lichen Material

*L. pulmonaria* (L.) Hoff. was collected from trunks of *Populus tremuloides* in the forest outskirts of Syktyvkar, Komi Republic, Russia (latitude 61°34′ N, longitude 50°33′ E). Thalli were placed between sheets of filter paper and left to air-dry slowly at room temperature for 2 d. Dried materials were stored at −20 °C until needed.

### 2.2. Stress Treatments

In our preliminary investigations, we found subjecting lichens to extreme temperatures of +40 °C and −20 °C for 3 h was the most effective in inducing a stress response [22]. For comparison, the effects of exposure to temperatures corresponding to more moderate natural seasonal temperatures that occur during the growing season (+4 °C and +30 °C) were studied. Before temperature treatment, dry thalli (n = 24) were prehydrated at +10 °C in a semi-transparent container on a damp cloth under a lid in dim laboratory lighting for 48 h. Thalli were then gently blotted with filter paper before the experiment. On the day of the experiment, the containers holding the lichens were transferred to a standard laboratory bench at room temperature under a lamp for 2–3 h to allow for adaptation. Afterward, the thalli (n = 6) in each treatment group were subjected to moderate temperatures of +4 °C and +30 °C for 3 h. They were placed in a semi-transparent container on a damp cloth under glass with a slit, positioned beneath a lamp providing 14–16 µmol m^−2^ s^−1^ of cool white light from an LED lamp (Model CXSSD-1001, Linhai Zhonguan electronic Technology Co., Ltd., Linhai City, China). For the severe temperature stress experiment, 12 lichen fragments were divided into two groups. One group (n = 6) was exposed to freezing temperatures of −20 °C, while the other (n = 6) was subjected to heat at +40 °C for 3 h in a temperature-controlled chamber (TSO-1/80 SPU thermostat, JSC “Smolensk SD and TBSCS”, Smolensk, Russia). As with the moderate treatments, the thalli were placed in a semi-transparent container on a damp cloth under glass with a slit, positioned under a lamp. Hydrated thalli maintained at room temperature served as controls.

### 2.3. Determination of Respiration Rate

The respiration rate of lichen thalli was assessed using the Warburg apparatus manometric technique [22]. Samples of lichen thalli (n = 3), weighing 500 mg, were placed in Warburg flasks containing 0.5 mL of distilled water and left to stabilize for 10 min at a constant temperature. Oxygen consumption was then monitored using pressure gauges every 60 min over a 1 h period at room temperature. To maintain a consistent CO_2_ concentration, 300 μL of 20% NaOH was added to the vessels side-arm during the process. Rates of respiration were expressed as μL of O_2_ consumed h^−1^ g^−1^ dry mass.

### 2.4. Measurement of Electrolyte Leakage

The electrolytes released from lichen tissues were quantified using a modification of the method of [29]. Fragments of lichen thalli (n = 5) were submerged in vials containing 8 mL of Milli-Q double-distilled water and incubated at room temperature in a thermostat for 30 min. Following incubation, the electrical conductivity of the solution (C1) was measured using an Ohaus ST3100C-B conductometer (Parsippany, NJ, USA). The total electrolyte content (C2) was determined by measuring the electrical conductivity of the same solution after disrupting cell membranes at 100 °C for 30 min. The membrane stability index (MSI) was calculated as follows: MSI = [1 − (C1/C2)] × 100%.

### 2.5. Chlorophyll Fluorescence

Chlorophyll *a* fluorescence of lichen thalli (n = 6) was measured using an FMS1+ fluorometer (Hansatech Instruments, King’s Lynn, UK). After a period of dark adaptation of at least 10 min, a flash of saturating light to measure the maximum photochemical efficiency of PSII, F_V_/F_M_, where F_M_ is maximum fluorescence and F_V_—variable fluorescence—or (F_M_ − F_0_), where F_0_—minimal fluorescence yield. An actinic light of 33 µmol m^−2^ s^−1^ was then turned on. After fluorescence efficiency decreased to stationary F_T_ level, a second saturating pulse was given to determine the maximum fluorescence yield (F_M′_) in the light-adapted state and to calculate the relative electron transport rate rETR (rETR = 0.5 × PAR × Φ_PSII_), where PAR is photosynthetically active radiation, and Φ_PSII_ is the effective quantum yield of photochemical reactions in PSII in the light, calculated as (F_M′_ − F_T_)/F_M_ [30].

### 2.6. Gene Identification and Retrieval of Protein Sequences

The complete genome of *L. pulmonaria* had been sequenced and deposited in the JGI databases for MycoCosm (The Fungal Genomics Resources: https://mycocosm.jgi.doe.gov/Lobpul1/Lobpul1.home.html, accessed on 19 October 2023) and PhycoCosm (The Algal Genomics Resources: https://phycocosm.jgi.doe.gov/Dicre1/Dicre1.home.html, accessed on 19 October 2023). To identify genes encoding squalene epoxidase from *L. pulmonaria* and *S. reticulata*, database searches were conducted using BLAST with *S. cerevisiae* and *C. reinhardtii* sequences as queries. The resulting sequences were then submitted to PFAM [31] and InterProScan [32] for verification in order to confirm the domain of the squalene epoxidase. The squalene epoxidase from mycobiont (*L. pulmonaria*) and photobiont (*S. reticulata*) were designated as LpSQE1 (corresponding transcript ID: 3806187) and SrSQE1 (corresponding transcript ID: 547185), respectively.

### 2.7. Sequence Analysis

The physicochemical properties of the SQE1 proteins, including molecular weight, isoelectric point, instability index, and grand average of hydropathicity (GRAVY), were predicted using the Expasy ProtParam tool [33]. The MULocDeep [34] was used to predict subcellular localization. Pairwise sequence alignment (PSA) was performed by Smith–Waterman local alignment using EMBOSS water (https://www.ebi.ac.uk/seqdb/confluence/display/JDSAT/Pairwise+Sequence+Alignment, accessed on 19 February 2024).

The MEME suite (http://meme-suite.org/index.html, accessed on 19 February 2024) was used to predict conserved motifs in SQE1 protein sequences. The analysis was conducted with the following parameters: zero or one site per sequence, number of motifs (1–10), motif width (6–50) [35]. Following the MEME analysis, the motif map was reconstructed using the TBtools-II (Toolbox for Biologists) v2.069 software [36].

The secondary structure of the SQE1 protein sequences was predicted by the online software NPSA (https://npsa-pbil.ibcp.fr/cgi-bin/npsa_automat.pl?page=/NPSA/npsa_sopma_f.html, accessed on 19 February 2024) [37], while the tertiary protein structure models were generated using the Robetta server (https://robetta.bakerlab.org, https://academic.oup.com/nar/article/32/suppl_2/W526/1040731?login=true, accessed on 23 February 2024). The visualization of the 3D models was carried out using Schrodinger Maestro software v2019-3. The alignment of the tertiary structures of mycobiont and photobiont squalene epoxidases was conducted in Chimera (https://onlinelibrary.wiley.com/doi/pdf/10.1002/jcc.20084, accessed on 19 February 2024).

### 2.8. Phylogenetic and Comparative Analyses

Homologous sequences of SQE1 proteins within the orders of lichenized ascomycetes *Caliciales, Graphidales, Lecanorales, Peltigerales*, and *Teloschistales* for the mycobiont, and classes of *Mamiellophyceae*, *Trebouxiophyceae*, and *Chlorophyceae* for the photobiont were obtained using BLASTP in the NCBI database. The sequences were aligned using the MUSCLE algorithm with default parameters. To remove gaps between the alignments, the aligned sequences underwent trimming using trimAl v1.2 software [38] with the “gappyout” setting. Subsequently, a phylogenetic tree was constructed using the maximum likelihood (ML) method in the IQ-TREE program [39] (http://iqtree.cibiv.univie.ac.at, accessed on 11 March 2024), with the following settings: “-st AA -m TEST -bb 1000 -alrt 1000”, and an ultra-fast bootstrap value of 1000 [40]. Finally, the iTOL tool was employed to visualize the resultant phylogenetic tree [41].

### 2.9. RNA Extraction, cDNA Synthesis, and RT-qPCR

Approximately five thalli fragments (n = 5) were collected immediately after the temperature stress treatments, quickly fixed in liquid nitrogen, and ground into a fine powder. For RT-qPCR analysis, 0.1 g of the powdered sample from each replicate was used. Using the RNeasy Plant Mini kit (Qiagen, Hilden, Germany) and an on-column DNase digestion step (DNase 1, QIAGEN), total RNA was extracted from *L. pulmonaria* thalli in accordance with the manufacturer’s instructions. A NanoDrop^®^ ND-1000 spectrophotometer (Thermo Fisher Scientific, Waltham, MA, USA) was used to determine the RNA concentrations and purity. The integrity of the sample was assessed using gel electrophoresis in a 1% (*w*/*v*) agarose gel. The Evrogen MMLV RT kit was used to synthesize cDNA according to the manufacturer’s protocols (Evrogen JSC, Moscow, Russia).

The vector NTI Suite 9 software was used to design RT-qPCR primers with the following parameters: amplicon length from 60 to 300 bp and a Tm range of 55 to 65 °C. RT-qPCR was performed on CFX Connect™ Real-Time System (Bio-Rad Laboratories, Singapore) with qPCRmix-HS SYBR (Evrogen). The templates were amplified three times at 95 °C for 3 min followed by 40 cycles of amplification (94 °C for 10 s and 55/60 °C for 40 s). The specificity of the primers was evaluated using a melting curve analysis following RT-qPCR and a gel electrophoresis analysis of the amplified products. The gene-specific primers used for RT-qPCR are listed in Table S1. α-ketoglutarate dehydrogenase (*LpKD*, Transcript ID: 859686), Pyruvate dehydrogenase (*LpPD*, Transcript ID: 4017210), Translation elongation factor 1a (*SrEF1*, Transcript ID: 599430), Pyruvate dehydrogenase (*SrPD*, Transcript ID: 64143) were used as reference genes for RT-qPCR normalization.

### 2.10. Statistical Analysis

Oxygen consumption was determined in three biological replicates and MSI in five biological replicates. Biological replicates were samples taken from different thalli. Chlorophyll fluorescence was detected in six biological replicates. Five biological and six analytical replicates were utilized for the RT-qPCR experiment. Gene expression differences were evaluated using normalized expression (Cq) in the Bio-Rad CFX MaestroTM/Software v2.3, and significance was determined for *p*-values ≤ 0.05 (*), *p* ≤ 0.01 (**), and *p* ≤ 0.001 (***) after ANOVA testing. The standard errors of the mean are depicted as vertical bars (n = 6). For the changes in oxygen consumption by *L. pulmonaria* thalli and MSI, the figure shows arithmetic means and standard errors (SE) for n = 5. The significance of the differences was also determined using ANOVA with (*) *p* ≤ 0.05, (**) *p* ≤ 0.01, and (***) *p* ≤ 0.001.

## 3. Results

### 3.1. Analysis of the Physiological Status of L. pulmonaria under Different Temperatures

Following the temperature treatments, the membrane stability index (MSI), respiratory activity of lichens, and chlorophyll fluorescence were measured to assess the physiological status of *L. pulmonaria*. The evaluation of lichen cell membrane permeability was conducted by measuring the release of electrolytes from the cells, which was used to calculate the MSI. Exposure to temperatures of −20 °C, +4 °C, and +30 °C did not result in noticeable changes in the MSI of thalli cells (see Table 1). However, subjecting lichen thalli to an elevated temperature of +40 °C markedly affected the MSI of *L. pulmonaria* cells.

After 1 h of exposure to low (+4 °C) or negative (−20 °C) temperatures, the respiration rate of *L. pulmonaria* was halved compared to the control (Table 1). Additionally, exposure to +40 °C significantly inhibited the rate of respiration. Chlorophyll fluorescence measurements showed that exposure to negative temperature (−20 °C), as well as temperatures of +4 °C and +30 °C, did not lead to significant changes in the maximum photochemical efficiency of PSII (F_V_/F_M_) and rETR (Table 1), while a low temperature of −20 °C reduced rETR. Exposure to +40 °C sharply suppressed these parameters.

### 3.2. Effects of Low and Elevated Temperatures on the Expression of Hsp Genes in the Symbionts of L. pulmonaria

**Figure 2:**
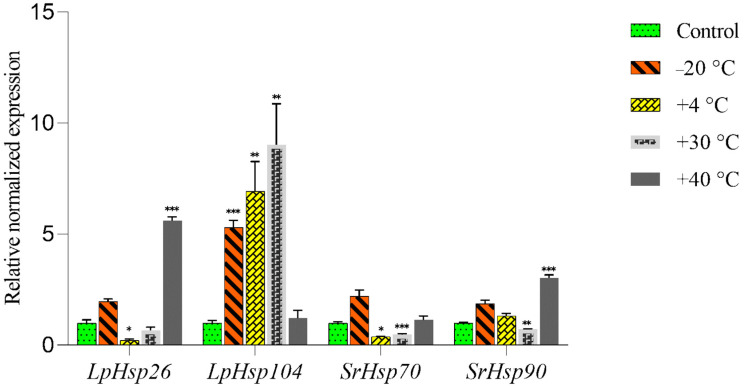
Relative expression of *LpHsp26*, *LpHsp104*, *SrHps70*, and *SrHsp90* genes assessed with RT-qPCR following exposure of thalli to freezing temperatures of −20 °C, +4 °C, +30 °C, and +40 °C for 3 h. Hydrated thalli kept at room temperature were used as controls. The significance of the differences was also determined using ANOVA with * *p* ≤ 0.05, ** *p* ≤ 0.01, and *** *p* ≤ 0.001.

Exposure to some temperatures significantly affected the expression of *Hsp* genes in *L. pulmonaria* (Figure 2).

Exposure to −20 °C did not significantly change the expression of *LpHsp26* in *L. pulmonaria*, but it did significantly increase the expression of *LpHsp104* (Figure 2). *SrHsp70* and *SrHsp90* from the photobiont both showed slight (non-significant) increases in expression after exposure to −20 °C. Expression of *LpHsp26* significantly decreased at +4 °C, while *LpHsp104* expression greatly increased (almost 7-fold) at this temperature. Exposing *L. pulmonaria* thalli to +30 °C also resulted in a significant increase in *LpHsp104* expression (9-fold). Conversely, the expression of *SrHsp70* was suppressed at both +4 °C and +30 °C, and *SrHsp90* expression was also downregulated at +30 °C. Exposure to +40 °C significantly increased *LpHsp26* expression, but did not affect the expression of *LpHsp104* (Figure 2). While +40 °C did not influence the expression of *SrHsp70*, it more than doubled *SrHsp90* expression.

### 3.3. Characterization and of SQE1 Genes Using Phylogenetic and Comparative Analyses

Squalene epoxidase 1 genes from *L. pulmonaria* and its green algal photobiont, *S. reticulata*, were retrieved from the JGI databases for MycoCosm and PhycoCosm, respectively. Analysis of the coding proteins using the databases PFAM [29] and InterProScan [30] revealed that the LpSQE1 and SrSQE1 investigated here have a squalene epoxidase domain structure. In addition, the length of the coding domain sequences (CDSs) (bp), the subcellular localization of LpSQE1 and SrSQE1, and various physicochemical properties such as protein length (aa), molecular weight (MW) kDa, isoelectric point (pIs), instability index, and GRAVY were predicted in silico (Table S2). The analyses indicated that these proteins are alkaline, with predicted pI values of 8.58 and 8.64 for LpSQE1 and SrSQE1, respectively. Furthermore, estimation of the instability indexes indicated that these proteins have unstable structures, with values above 40. Positive values of GRAVY showed that LpSQE1 and SrSQE1 were hydrophobic, and subcellular localization prediction suggested that both proteins are localized in the endoplasmic reticulum.

**Figure 3:**
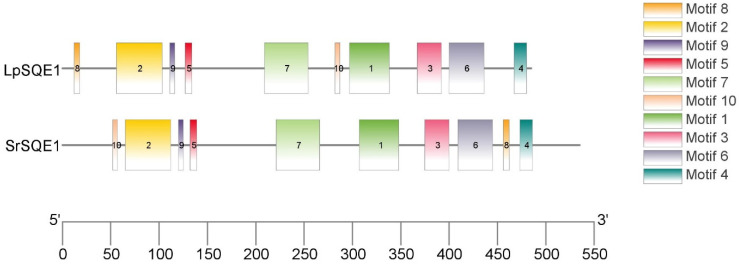
Distribution of 10 putative conserved motifs within the LpSQE1 and SrSQE1 proteins. Each conserved motif is represented by a differently colored box.

Pairwise sequence comparison revealed that LpSQE1 and SrSQE1 amino acid sequences share 55.2% similarity. Additionally, motif analysis using MEME Suite (Figure 3) identified 10 conserved motifs in both LpSQE1 and SrSQE1.

While motifs 8 and 10 are present in both proteins, they occur in different positions within the amino acid sequences. For example, in LpSQE1, motif 8 appears at the beginning of the sequence, whereas motif 10 is positioned in the middle of the sequence immediately after motif 7. Conversely, SrSQE1 begins with motif 10, and motif 8 appears just before motif 4 at the end of the sequence. The presence of conserved motifs in both LpSQE1 and SrSQE1 suggests that these motifs may play important functional roles in the proteins.

The secondary structures of the LpSQE1 and SrSQE1 proteins both consist of four structural patterns: α-helix, extended strand, β-turn, and random coil (Table S3). Each of the four structures contains a different number of amino acids. The random coil (Cc) and α-helix (Hh) components make up the majority of the secondary structure of the SQE1 proteins. Furthermore, the tertiary structure of the proteins indicated that they have very similar tertiary structures, including conserved domains and overall organization (Figure S1A,B). The two tertiary structures of SQE1 from the mycobiont and photobiont exhibited a remarkably conserved structural overlap, with minimal variation in their conformation (Figure S1C).

**Figure 4:**
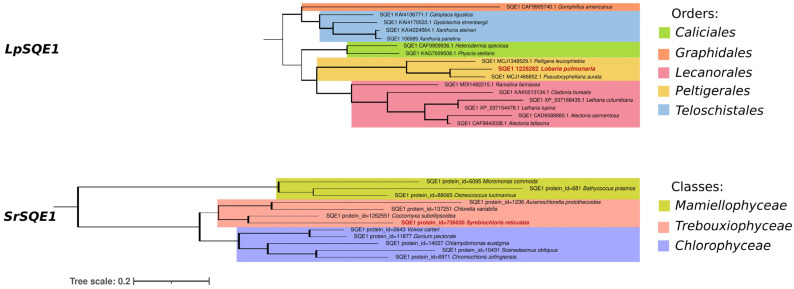
Phylogenetic tree constructed from the LpSQE1 and SrSQE1 protein sequences using the maximum likelihood (ML) method in IQ-TREE, illustrating the relationship between the amino acid sequences of LpSQE1 and SrSQE1 and squalene epoxidase protein sequences from class *Lecanoromycetes* and phylum *Chlorophyta*. The ML tree and bootstrap support values are based on 1000 replicates. Bold branches indicate bootstrap support ≥90. The scale represents 0.2 amino acid sequence substitutions per site. Proteins encoded by genes of *L. pulmonaria* and *S. reticulata* are marked in bold and red. Branches are colored according to the taxonomy of organisms. Please note the correct name for *Pseudocyphellaria aurata* is *Crocodia aurata*.

Figure 4 illustrates the phylogenetic tree constructed using 15 SQE1 protein sequences from other mycobionts within the orders *Caliciales*, *Graphidales*, *Lecanorales*, *Peltigerales*, and *Teloschistales*, along with 11 SQE1 protein sequences from photobionts within the classes *Mamiellophyceae*, *Trebouxiophyceae*, and *Chlorophyceae* obtained from NCBI.

Phylogenetic analyses reveal two specifically labeled trees: LpSQE1 for *L. pulmonaria* and SrSQE1 for *S. reticulata*. According to the results, both LpSQE1 and SrSQE1 trees are divided into two main clades with several subgroups, indicating further genetic diversification within each group. Apparently, LpSQE1 clustered together with SQE1s from *Peltigera leucophlebia* and *Crocodia aurata* within the order Peltigerales, suggesting a common ancestry among these proteins. Similarly, SrSQE1 is grouped with SQE1s from *Coccomyxa subellipsoidea*, *Chlorella variabilis*, and *Auxenochlorella protothecoides* within the class Trebouxiophyceae, indicating fewer evolutionary changes since their divergence from a common ancestor.

### 3.4. Effects of Low and Elevated Temperatures on the Expression of SQE1 Genes in the Symbionts of L. pulmonaria

**Figure 5:**
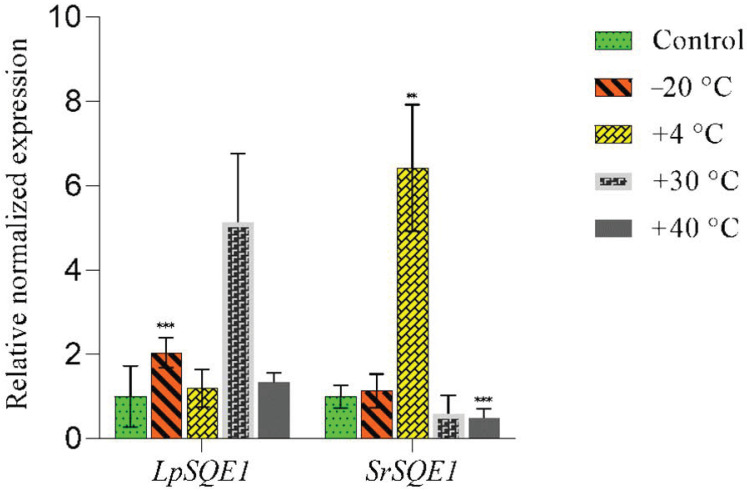
Relative expression of *LpSQE1* and *SrSQE1* genes assessed with RT-qPCR when thalli were exposed to freezing temperatures of −20 °C, +4 °C, +30 °C, and +40 °C for 3 h. Hydrated thalli kept at room temperature were used as controls. The significance of the differences was also determined using ANOVA with ** *p* ≤ 0.01, and *** *p* ≤ 0.001.

Exposing lichen thalli to freezing, cold, and moderate and elevated temperatures changed the expression levels of *LpSQE1* and *SrSQE1* (Figure 5). Under freezing conditions (−20 °C), *LpSQE1* expression in *L. pulmonaria* thalli was upregulated. However, *SrSQE1* expression remained largely unchanged at this temperature.

Exposure to a chilling temperature (+4 °C) did not alter *LpSQE1* expression. However, subjecting thalli to +30 °C upregulated *LpSQE1* expression. Conversely, *SrSQE1* expression increased at +4 °C, but slightly decreased at +30 °C. While heat stress (+40 °C) did not significantly affect *LpSQE1* expression, it reduced *SrSQE1* expression. These findings suggest that *LpSQE1* and *SrSQE1* may play distinct roles in the response of the different symbionts to temperature stresses.

## 4. Discussion

Species worldwide have faced challenges adapting to changing environments throughout evolutionary periods, but anthropogenic climate change has caused unprecedented temperature shifts in most ecosystems [42,43]. A recent modeling study indicated that lichens would need to migrate impossibly fast to maintain their current temperature optima, implying that extinctions may become common [14]. Due to the consequences of climate change, the decline in the populations of the lichen *L. pulmonaria* is expected to accelerate in the foreseeable future, because changes in the environment are likely to exceed the buffering capacity of this species, i.e., its ecological adaptability [44]. In our study, we investigated the effects of a range of temperatures on the thalli of the lichen *L. pulmonaria*. Temperatures of +4 °C and +30 °C are regularly experienced in the field by this lichen, whereas temperatures of −20 °C and +40 °C can be considered more extreme. While only very limited information is available about the ways in which lichens tolerate adverse temperatures, the results presented here indicate that sterol metabolism may play an important role, as has been reported in other organisms [22].

Membrane permeability is a sensitive indicator for assessing the physiological response of lichens to environmental stimuli [45]. In the present study, the decrease in the MSI observed after exposure of *L. pulmonaria* thalli to +40 °C suggests a disruption in membrane integrity and a state of stress for the lichen (Table 1). This decrease in the MSI was accompanied by an inhibition of respiration. We recently documented the reduction in the MSI that occurs following heat stress in *P. canina* thalli, which experienced a decrease in the MSI following exposure to adverse temperatures [22].

Several studies have documented the effect of environmental conditions on the respiration rates of lichens [46,47]. In general, respiration increases with an increase in temperature [6,47], such that an increase of 10 °C causes a 2- to 3-fold rise in the rate of respiration [48,49]. Here, exposing thalli to low temperatures, namely +4 °C and −20 °C, significantly reduced respiration (Table 1). Similar results were obtained by [50], where a decrease in temperature from 35 °C to 5 °C suppressed the respiratory activity of the lichen *Usnea*. Studies have shown that lichens can carry out respiration and photosynthesis at temperatures as low as −12 °C and −24 °C, respectively [51]. We recently showed that respiration was inhibited at elevated temperatures in *P. canina* thalli [22], consistent with earlier findings [52]. It has been reported that lichens display an initial and rapid increase in respiration within the first 15 min after a temperature rise of 5 °C, followed by a gradual decrease [53]. In contrast, for PSII activity, within the timeframe of our experiments, F_V_/F_M_ and rETR were only significantly inhibited by +40 °C. Taken together, these results suggest that +40 °C damages both the mycobiont and the photobiont of the lichen.

An increasing number of studies have used gene expression to study the response of organisms to environmental stress [54,55]. In free-living fungi, high temperatures can potentially affect numerous physiological processes, including growth. Hsps, or stress-induced proteins, play a crucial role in repairing or eliminating heat-induced cellular damage by preventing the aggregation of denatured proteins [56]. Here, we investigated the effects of low and elevated temperatures on the expression of *Hsp* genes in the symbionts of *L. pulmonaria*. Our results revealed that different *Hsps* responded differently to various temperatures. *LpHsp26* decreased at +4 °C, while *LpHsp104* increased at both +4 °C and +30 °C. Moreover, *SrHsp70* increased slightly at −20 °C, but not at +40 °C, while *SrHsp90* was induced by both extreme temperatures. This suggests that lichen thalli respond to extreme temperatures by adjusting *Hsp* expression to protect cells and cope with stress. Overall, these findings indicate that *Hsp* genes are likely to play important roles in *L. pulmonaria’s* response to temperature stress, assisting the lichen to adapt to a range of environmental conditions.

Other studies have also shown that in *L. pulmonaria* and its photobiont *S. reticulata,* heat shock genes are upregulated as temperatures increase from 4 to 15 °C and from 15 to 25 °C [17]. This finding is consistent with several studies reporting increased expression of molecular chaperone genes, such as *Hsps*, in response to temperature increases in fungi, including lichenized species [57,58]. A study [5] showed increased expression of Hsp88 and Hsp98 in *L. pulmonaria* at moderate and high temperatures (15 and 25 °C), suggesting that moderate temperature changes can induce heat shock reactions in *L. pulmonaria*, although the effect was more pronounced at 25 °C compared to 15 °C. Furthermore, previous research by [17] revealed that there was an increase in the expression of *Hsps* in both the mycobiont and photobiont of *L. pulmonaria* exposed to sudden temperature changes. These *Hsps* are essential for cellular protection, as they help to repair or remove damage caused by heat stress by preventing the aggregation of denatured proteins [18,19]. Our results highlight the dynamic regulation of *Hsp* genes in *L. pulmonaria* under various temperature conditions, potentially indicating an adaptive response to withstand heat stress.

Cells can perceive temperature signals through changes in the membrane state [59]. Regulation of the membrane state, particularly its fluidity, occurs through changes in membrane components, including sterols and triterpenoids. It has been suggested that squalene epoxidase, an enzyme of the mevalonate pathway biosynthesizing these membrane components, may play a crucial role in regulating membrane fluidity under temperature stress conditions [22].

In this study, using an in silico approach, we report on the identification and molecular characterization of the cDNAs that encode squalene epoxidase in *L. pulmonaria* and its photobiont *S. reticulata*. The physicochemical analysis revealed that LpSQE1 and SrSQE1 are alkaline proteins with *pI* values of 8.58 and 8.64, respectively (Table S2), and subcellular localization prediction indicates that LpSQE1 and SrSQE1 are located in the endoplasmic reticulum (ER) (Table S2). Yeast SQE1 (erg1p) is localized in the ER and lipid droplets. Endoplasmic reticulum proteins are essential for squalene cyclooxygenase activity [36]. In yeast, squalene epoxidase facilitates the association between lipid molecules and the ER [60,61]. Reports from the fungi are consistent with those from higher plants, e.g., the *SQE1* gene in *Dioscorea zingiberensis* has been shown to be localized in the ER [61]. Additionally, *BsSE1* from *Bletilla striata* has been shown to localize to the ER membrane [60]. It seems likely that the ER provides suitable conditions for the completion of the squalene epoxidase oxidation reaction [62].

Furthermore, despite coming from different Phyla, sequence alignment suggests that LpSQE1 and SrSQE1 are clearly related proteins with a certain degree of sequence similarity. Analysis with MEME Suite identified 10 conserved motifs in both LpSQE1 and SrSQE1. However, the positional distribution of motifs 8 and 10 differed between the two proteins (Figure 3), which may indicate some differences in their functions or regulatory mechanisms. Despite these differences, the presence of the same conserved motifs in both LpSQE1 and SrSQE1 suggests that these motifs play an important functional role in these proteins.

Interestingly, structural analysis revealed that the LpSQE1 and SrSQE1 proteins possess similar tertiary structures with conserved domains (Figure S1). Furthermore, their secondary structures predominantly consist of random coils and α-helices, as shown in Table S3. The secondary structure of a protein can influence its function. For example, α-helices are often involved in protein–protein interactions and contribute to structural stability, particularly with respect to thermal stability [63]. Conversely, random coils contribute to increased protein flexibility and facilitate conformational changes [64]. Nevertheless, the overall structural similarity, especially in their tertiary structures, may indicate evolutionary conservation or similar functions between the LpSQE1 and SrSQE1 proteins.

Phylogenetic analysis of the SQE1 protein sequences from mycobionts and photobionts provides valuable insights into the evolutionary relationships among these organisms (Figure 4). The clustering of SQE1 proteins from *L. pulmonaria* (LpSQE1) with those from *P. leucophlebia* and *P. aurata* that are also in the order *Peltigerales* indicate a close evolutionary relationship and reflect the common ancestry of these proteins. As would be expected, this clustering indicates that these organisms may share a more recent common ancestor compared to other sequences included in the analysis. Similarly, the grouping of SQE1 proteins from *S. reticulata* (SrSQE1) with those from *C. subellipsoidea*, *C. variabilis*, and *A. protothecoides* within the class *Trebouxiophyceae* suggests a common evolutionary history (Figure 4). The close clustering of these sequences implies few evolutionary changes have taken place since their divergence from a common ancestor, indicating that there is a highly conserved genetic makeup among these organisms.

Products of the mevalonate pathway, including sterols and triterpenes, may contribute to the formation of a stress response to temperature exposure. Lichens are known to have a very rich spectrum of triterpenes, with around 90 triterpene-like compounds identified in lichens [65]. Most plants, fungi, animals, and lichens produce the triterpene squalene as a biochemical precursor in sterol biosynthesis. Squalene, along with cyclic triterpenes and sterols, is necessary for the homeostasis of permeability and fluidity of eukaryotic membranes [66]. A crucial step in the mevalonate pathway, common to all organisms, is the oxygenation of squalene to form 2,3-oxidosqualene, catalyzed by the enzyme squalene epoxidase, which determines the levels of sterols and triterpenes [20].

Squalene epoxidase is therefore a major regulator of the sterol biosynthesis pathway and plays a key role in fungi, contributing to photooxidation through the production of ergosterol [67]. The activation of this enzyme requires oxygen, nicotinamide adenine dinucleotide phosphate (NADP^+^), hydrogen, and flavin adenine dinucleotide (FAD) [68]. Notably, SQE1 in the free-living basidiomycete *Ganoderma lucidum* stimulates the accumulation of ganoderic acid and enhances the expression of the lanosterol synthase gene in the biosynthetic pathway [23]. Stable expression of *GgSQE1* from the higher plant *Glycyrrhiza glabra* can regulate the sterol content in tobacco [24]. *SQE1* also plays a role in plant growth and development. For example, in *Arabidopsis*, SQE1 is critical for plant growth, affecting root length and hypocotyl elongation [68]. Furthermore, *ArSQE* plays a significant role in the early germination of transgenic tobacco. Transgenic tobacco seedlings overexpressing *ArSQE* demonstrate tolerance to abiotic stress [26]. Squalene epoxidase is considered a rate-limiting enzyme as it serves as a key control point in the sterol biosynthesis pathway [24,25]. Squalene epoxidase is encoded by the *SQE1* (*ERG1*) gene, which has been cloned and characterized in various organisms such as *S. cerevisiae*, *Candida albicans*, *Aspergillus fumigatus*, *Medicago truncatula*, rats, and humans [67,69]

However, the present study is the first to characterize the molecular properties and temperature stress-related gene expression of *SQE1* in the mycobiont of *L. pulmonaria* and its algal photobiont, *S. reticulata*. An important aspect of our study was to investigate the specificity of the stress response of both the mycobiont and the photobiont, mediated by the activity of the *SQE1* gene, under adverse temperature conditions.

In the present study, RT-qPCR analyses indicated that expression of the *LpSQE1* and *SrSQE1* genes was strongly temperature-dependent (Figure 5). At a low positive temperature (+4 °C), *SrSQE1* expression significantly increased in the photobiont, while *LpSQE1* expression in the mycobiont remained unchanged. At +30 °C, *LpSQE1* expression increased in the mycobiont, with little effect on *SrSQE1*. At an extreme temperature of +40 °C, the *SrSQE1* levels decreased, and *LpSQE1* expression remained unchanged (Figure 5). Freezing to −20 °C increased *LpSQE1* expression but had little effect on *SrSQE1*. The results are consistent with those of [28], who reported the upregulation of ERG genes at 25 °C in the eukaryotic photobiont of *Peltigera britannica*. The strong dependence of SQE1 expression on temperature suggests that both genes play important roles in the response of *L. pulmonaria* thalli to this stress.

## 5. Conclusions

This study examined the responses of *L. pulmonaria* to freezing, cold, and high temperatures by analyzing respiratory activity, chlorophyll α fluorescence, the membrane stability index (MSI), and gene expression. Exposure to temperatures of +4 °C and +30 °C does not cause noticeable changes in the respiration rate, MSI, or chlorophyll α fluorescence parameters (F_V_/F_M_ and rETR), suggesting that in the short term at least, these temperatures are not stressful for *L. pulmonaria* and fall within the range of normal temperature fluctuations to which the lichen is well adapted. However, an increase in temperature of 10 °C to +40 °C greatly reduced respiration, PSII activity, and membrane stability. Furthermore, the expression of *Hsp* and *SQE1* genes was strongly temperature-dependent, suggesting that lichen thalli respond to extreme temperatures by adjusting the expression of these genes to protect cells and cope with stress. The changes that occur in the expression of SQE1 genes, identified in silico in the mycobiont and photobiont of *L. pulmonaria* when exposed to different temperatures, may indicate that SQE1 in each of the symbionts plays a unique role in the lichen’s overall response to temperature stress. Taken together, our results add support to the growing evidence that sterols play important roles in stress tolerance in all organisms [22].

## Figures and Tables

**Table 1:** The membrane stability index, rates of respiration, and photosynthesis of *L. pulmonaria* lichen after temperature treatment.

Samples	MSI, %	Respiration Rate, µL/h/g Dry Mass	Photosynthesis	
			F_V_/F_M_	rETR
Control	97.5 ± 0.4	422 ± 26	0.71 ± 0.0	7.2 ± 0.2
−20 °C	94.9 ± 0.1 **	198 ± 65 ***	0.70 ± 0.0	5.2 ± 0.9
+4 °C	94.9 ± 0.2 **	186 ± 35 ***	0.71 ± 0.0	7.3 ± 0.3
+30 °C	96.0 ± 0.2 *	361 ± 88	0.71 ± 0.0	7.4 ± 0.3
+40 °C	78.2 ± 0.2 ***	97 ± 16 ***	0.27 ± 0.0 ***	0.4 ± 0.1 ***

The table shows the arithmetic mean values and standard errors (SE) The significance of the differences was also determined using ANOVA with * *p* ≤ 0.05, ** *p* ≤ 0.01, and *** *p* ≤ 0.001.

## Data Availability

The original contributions presented in the study are included in the article/Supplementary Material, further inquiries can be directed to the corresponding author.

## Supplementary Materials

The following supporting information can be downloaded at: https://www.mdpi.com/article/10.3390/jof10100705/s1, Figure S1: tertiary structure of (A) LpSQE1 and (B) SrSQE1 showing their structural organization. The models presented are those with the highest sequence identity and alignment coverage obtained from Robetta server. Protein chains are displayed as solid ribbons. Alignment of tertiary structures of mycobiont (LpSQE1—magenta color) and photobiont (SrSQE1—green color) (C) was performed in Chimera; Table S1: primers of RT-qPCR; Table S2: physicochemical properties and subcellular localization of LpSQE1 and SrSQE1; Table S3: protein secondary structure: α-helix, extended strand, β-turn, and random coil data.
