# Supplementary material for: Role of Squalene Epoxidase Gene (SQE1) in the Response of the Lichen Lobaria pulmonaria to Temperature Stress
Source: J Fungi (Basel). 2024 Oct 9;10(10):705. doi: 10.3390/jof10100705 (PMC11508302; doi:10.3390/jof10100705)
Supplement: Supplementary file 1 [file jof-10-00705-s001.zip › Figure S1.pdf]

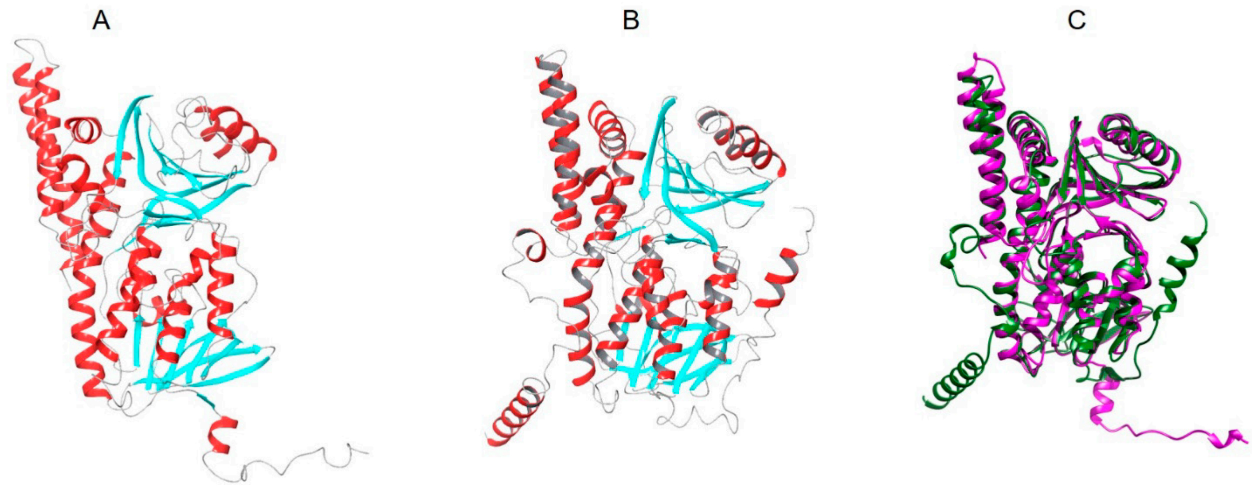

Figure S1. Tertiary structure of (**A**) LpSQE1 and (**B**) SrSQE1 showing their structural organization. The models presented are those with the highest sequence identity and alignment coverage obtained from Robetta server. Protein chains are displayed as solid ribbons. Alignment of tertiary structures of mycobiont (LpSQE1 – magenta color) and photobiont (SrSQE1 – green color) (**C**), was performed in Chimera.
