# Supplementary material for: Role of Squalene Epoxidase Gene (SQE1) in the Response of the Lichen Lobaria pulmonaria to Temperature Stress
Source: J Fungi (Basel). 2024 Oct 9;10(10):705. doi: 10.3390/jof10100705 (PMC11508302; doi:10.3390/jof10100705)
Supplement: Supplementary file 1 [file jof-10-00705-s001.zip › Table S1.pdf]

Table S1. Primers of RT-qPCR.

Gene name	Transcript ID	Forward primer (5′–3′)	Reverse primer (5′–3′)	Product size (bp)	Annealing temp (ºC)
LpSQE1	3806187	CTTGCCATCACGCTCGCTC	GTTATTTCCACCCCCTCTCCATAG	210	56
SrSQE1	547185	CGGTCATACAGCATCCCTCCTAC	GCATCGCCCAGCAGCAG	350	56
LpKD	859686	GCAACTCAACCCTTTCCCCTGG	CTCGCCTTCAATCCCGTCG	229	60
LpPD	4017210	CACCCTCACTCCTTGCTCTTGC	GTCCATCTGTGCCTTGTCCGTTTC	192	60
SrEF1	599430	CAGGTGGAGAGATTGACGACG	CTTGGCACGAATGACAGCAG	84	60
SrPD	64143	GTAAGGTATCAGAAGGACGCAGTAGG	CATCAACAAGCACGCCAGG	273	55
LpHSP26	4184579	CAGCAACAGCCAACACTTCCTC	CATAGTCTTCGTCCTCCACCGTC	320	60
LpHSP104	4173914	CAGGAGCCAAGTTTAGGGGTG	CGAGAGCGGGTTTTAGAAGGTTAC	172	60
SrHSP70	446234	CTCACAGCGGCAAGCAACC	GTCAAAGTCCTCACCACCCAAG	253	60
SrHSP90	541741	CCCTCATCACCTCCAAGTTCG	GTCATCGCCTCAGCCTCCTC	190	60
