# Supplementary material for: Role of Squalene Epoxidase Gene (SQE1) in the Response of the Lichen Lobaria pulmonaria to Temperature Stress
Source: J Fungi (Basel). 2024 Oct 9;10(10):705. doi: 10.3390/jof10100705 (PMC11508302; doi:10.3390/jof10100705)
Supplement: Supplementary file 1 [file jof-10-00705-s001.zip › Table S2.pdf]

Table S2. Physico-chemical properties and subcellular localization of LpSQE1 and SrSQE1.

	Gene ID	CDS length (bp)	No. of amino acid (aa)	Molecular weight (kDa)	Isoelectric point	Instability index	GRAVY	Subcellular localization prediction
**Mycobiont (*L. pulmonaria*)**	*LpSQE1*	1455	484	53.2	8.58	51.56 (unstable)	+0.137	Endoplasm. retic.
**Photobiont (*S. reticulata*)**	*SrSQE1*	1605	534	57.9	8.64	47.42 (unstable)	+0.078	Endoplasm. retic.
