# Supplementary material for: Role of Squalene Epoxidase Gene (SQE1) in the Response of the Lichen Lobaria pulmonaria to Temperature Stress
Source: J Fungi (Basel). 2024 Oct 9;10(10):705. doi: 10.3390/jof10100705 (PMC11508302; doi:10.3390/jof10100705)
Supplement: Supplementary file 1 [file jof-10-00705-s001.zip › Table S3.pdf]

Table S3. Protein secondary structure: α-helix, extended strand, β-turn and random coil data

Protein ID	No. of amino acid	Hh (%) α-Helix	Ee (%) Extended strand	Tt (%) β-Turn	Cc (%) Random coil
LpSQE1	484	38.43	16.94	5.37	39.26
SrSQE1	534	35.77	15.36	6.18	42.70
